# How to address the coronaries in TAVI candidates: can the need for revascularization be safely determined by CT angiography only?

**DOI:** 10.1093/ehjimp/qyae096

**Published:** 2024-10-28

**Authors:** Katharina Theresa Julia Mascherbauer, Gudrun Lamm, Andreas Anselm Kammerlander, Maximilian Will, Christian Nitsche, Roya Anahita Mousavi, Caglayan Demirel, Philipp Emanuel Bartko, Konstantin Schwarz, Christian Hengstenberg, Julia Mascherbauer

**Affiliations:** Department of Internal Medicine 2, Division of Cardiology, Medical University of Vienna, Vienna, Austria; Department of Internal Medicine 3, University Hospital St. Pölten, Karl Landsteiner University of Health Sciences, Dunant-Platz 1, 3100 Krems, Austria; Department of Internal Medicine 2, Division of Cardiology, Medical University of Vienna, Vienna, Austria; Department of Internal Medicine 3, University Hospital St. Pölten, Karl Landsteiner University of Health Sciences, Dunant-Platz 1, 3100 Krems, Austria; Department of Internal Medicine 2, Division of Cardiology, Medical University of Vienna, Vienna, Austria; Department of Internal Medicine 3, University Hospital St. Pölten, Karl Landsteiner University of Health Sciences, Dunant-Platz 1, 3100 Krems, Austria; Department of Internal Medicine 2, Division of Cardiology, Medical University of Vienna, Vienna, Austria; Department of Internal Medicine 2, Division of Cardiology, Medical University of Vienna, Vienna, Austria; Department of Internal Medicine 3, University Hospital St. Pölten, Karl Landsteiner University of Health Sciences, Dunant-Platz 1, 3100 Krems, Austria; Department of Internal Medicine 2, Division of Cardiology, Medical University of Vienna, Vienna, Austria; Department of Internal Medicine 3, University Hospital St. Pölten, Karl Landsteiner University of Health Sciences, Dunant-Platz 1, 3100 Krems, Austria

**Keywords:** coronary angiography, computed tomography, TAVI, percutaneous coronary intervention

## Abstract

Coronary artery disease (CAD) remains one of the most frequent comorbidities among transcatheter aortic valve implantation (TAVI) candidates. Whether routine assessment of CAD by invasive coronary angiography (CA) and eventual peri-procedural percutaneous coronary intervention (PCI) is generally beneficial in TAVI patients has recently been heavily questioned. CA carries significant risks, such as kidney injury, bleeding, and prolonged hospital stay, and may frequently be unnecessary if significant stenoses of the proximal coronary segments can be ruled out on computed tomography angiography. Moreover, the benefits of pre-emptive coronary revascularization at the time of TAVI are not well defined. Despite these facts and weak guideline recommendations, CA and eventual PCI of stable significant coronary lesions at the time of TAVI remain common practice. However, ongoing randomized trials currently challenge the efficacy of such strategies to enable a more streamlined, individualized, and resource-sparing treatment with TAVI.

## Central Illustration

**Central Illustration:**
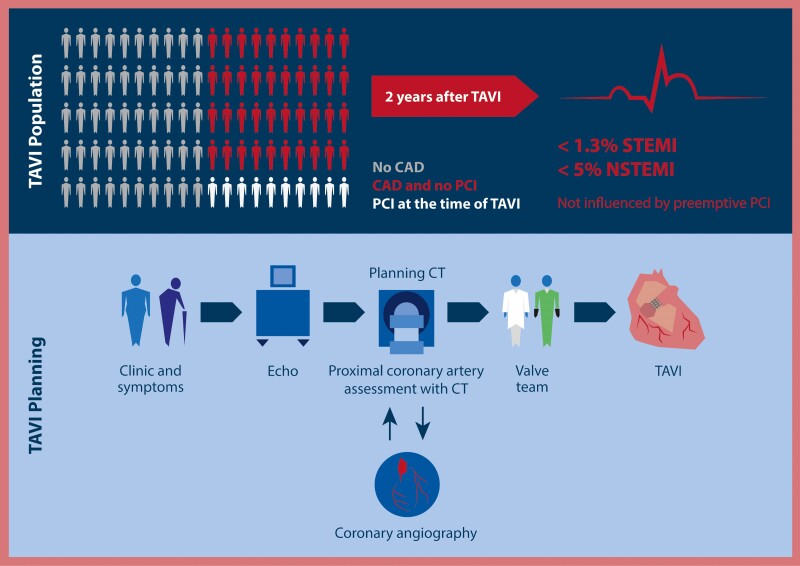
Prevalence of CAD and coronary revascularization in TAVI candidates (top). Overall, 50% of contemporary patients evaluated for TAVI present with concomitant CAD. Of these, 5–13% undergo PCI at the time of TAVI. The risk for acute coronary syndrome at 2 years after TAVI is low (NSTEMI < 5%; STEMI < 1.3%) and not influenced by pre-emptive PCI. Proposed TAVI planning path (bottom). Patients undergo clinical assessment, echocardiography, and a TAVI CT scan. All these assessments can be performed on an outpatient basis. We propose to assess the coronary arteries using the TAVI CT and perform invasive coronary angiography only, if proximal stenoses are detected on CT. CAD, coronary artery disease; CT, computed tomography; NSTEMI, non-ST-elevation myocardial infarction; PCI, percutaneous coronary intervention; STEMI, ST-elevation myocardial infarction; TAVI, transcatheter aortic valve implantation.

## Introduction

**Figure 1:**
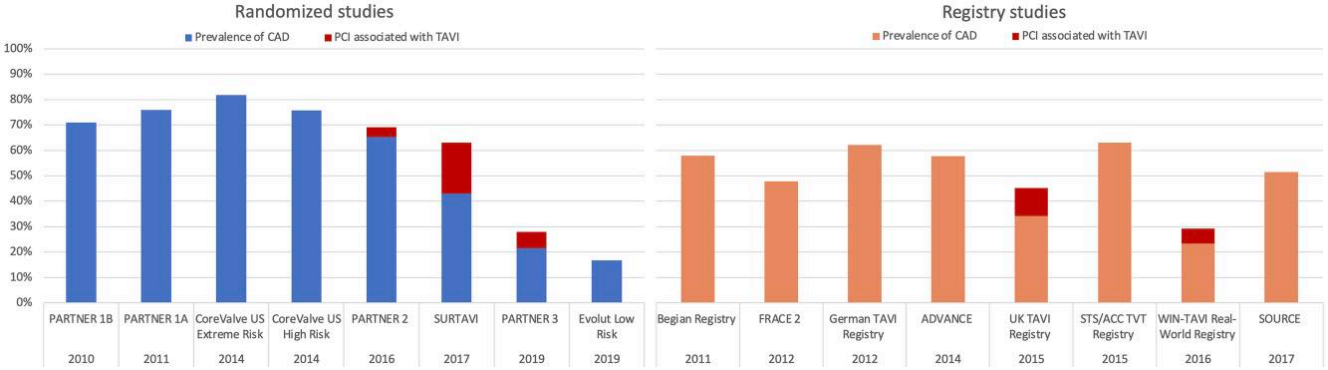
Prevalence of CAD in randomized studies and registries. Prevalence of CAD as reported in the major TAVI studies and registries of the last 14 years. CAD coronary artery disease; TAVI, transcatheter aortic valve implantation.

Coronary artery disease (CAD) and degenerative aortic stenosis (AS) are two distinct entities that often coexist. Up to 50% of contemporary AS patients undergoing evaluation for transcatheter aortic valve implantation (TAVI) are affected by CAD (*Central Illustration* and *Figure 1*).^1^ It is widespread routine practice to evaluate the coronary arteries by invasive coronary angiography (CA) during workup for TAVI and eventually intervene on significant CAD using percutaneous coronary intervention (PCI) before, during, or after TAVI.^1^ However, PCI of concomitant stable CAD remains of unclear impact on outcomes in TAVI patients.^2–7^

Several studies, including two meta-analyses, found no clinical benefit of pre-emptive coronary revascularization of stable CAD in TAVI patients.^2,3,6,8–10^ Moreover, a higher incidence of acute kidney injury and major bleeding was reported in patients who underwent PCI before or shortly after TAVI compared with those whose CAD was treated medically.^2^ On the contrary, incomplete revascularization of TAVI recipients [residual SYNTAX score (rSS) of >8] was, in a historical series, found to be related to increased mortality.^4,5^ The latest trial on the topic, NOTION-3, reported no impact on mortality related to pre-emptive revascularization of significant CAD at the time of TAVI but was positive with regard to reduction of urgent revascularization at follow-up.^3^

The practice of complete revascularization at the time of aortic valve replacement does not emanate from randomized-controlled data. It goes back to the common practice of treating CAD at the time of open-heart valve surgery.^6,9^ In the surgical setting, this approach is the mainstay since a second, foreseeable heart surgery should always be avoided, and the peri- and post-operative course may be compromised in non-revascularized patients. Following these principles of cardiac surgery, TAVI operators have, from the start, carefully assessed the severity of CAD prior to valve intervention. Such an approach was further supported by the concern that non-revascularized patients might not tolerate the drop in blood pressure during rapid pacing, which is frequently necessary during catheter-based valve implantation. Furthermore, coronary access may be more difficult after TAVI, particularly in prostheses with supra-annular design and narrow stent struts.^11–14^

The current European Society of Cardiology (ESC) guidelines for the management of valvular heart disease recommend that coronary revascularization should be considered before TAVI (Class IIa C).^15^ However, as indicated by the ‘C’, this recommendation relies on expert consensus, leaving considerable room for individual decision. In addition, uncertainty exists concerning the necessary extent of coronary revascularization in this setting.^4^ The 2020 American Heart Association (AHA) /American College of Cardiology (ACC) valve guidelines did not include recommendations regarding coronary revascularization at the time of TAVI.^16^

The aim of this article was to address the question of whether pre-emptive CA is indicated in every patient planned for TAVI and under which circumstances invasive CA can be safely omitted. In addition, we searched the current literature to clarify the proven benefits of pre-emptive PCI of stable CAD in TAVI recipients and addressed the question of whether an individualized assessment of the coronary arteries based on computed tomography angiography (CTA) would be safe and more resource efficient in selected patients.

## Assessment of CAD with CT in TAVI candidates

**Figure 2:**
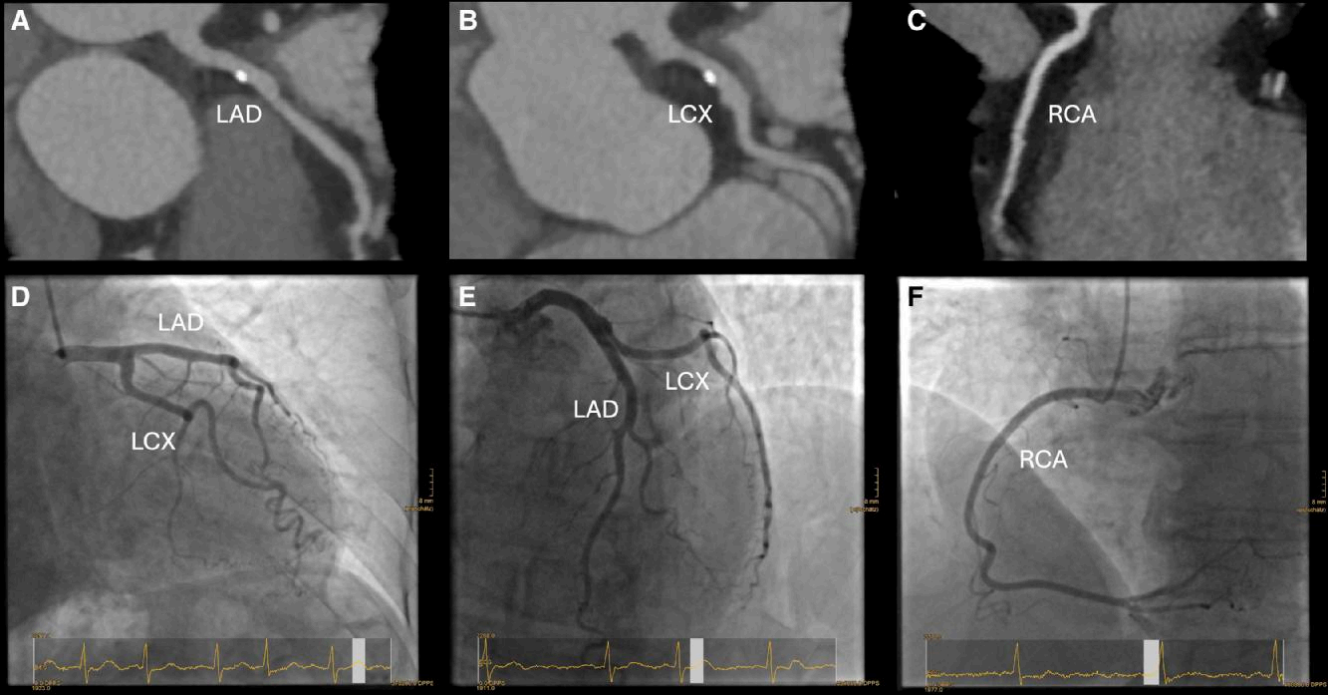
CTA (TAVI planning CT) and corresponding invasive CA and no coronary disease. A 84-year-old female patient, dyspnoea on exertion New York Heart Association (NYHA) III, no angina pectoris, and impaired kidney function. High-gradient AS on echocardiography with a peak velocity of 4.2 m/s. *A* shows the LAD, *B* shows the LCX, and *C* shows the RCA, all from the TAVI planning CT. *D–F* depict the corresponding images of stenosis-free coronaries by invasive CA. CT, computed tomography; LAD, left anterior descending artery; LCX, circumflex artery; RCA, right coronary artery; TAVI, transcatheter aortic valve implantation.

**Figure 3:**
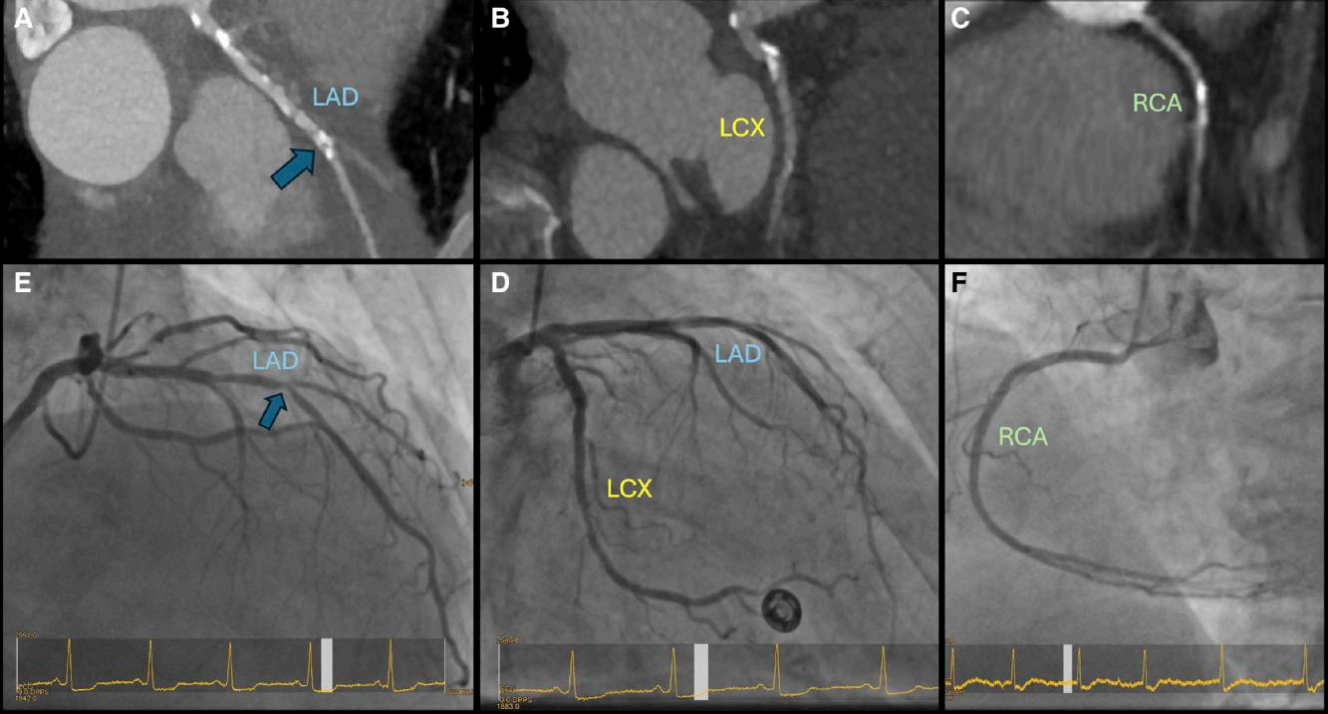
CTA (TAVI planning CT) and corresponding invasive CA and significant coronary disease. A 86-year-old male patient, dyspnoea on exertion NYHA III, no angina pectoris, and impaired kidney function. High-gradient AS, peak velocity on echocardiography 4.1 m/s. *A* shows left main stem and LAD with significant calcification and stenosis (blue arrow), *B* shows the LCX, which is also calcified, and *C* shows the RCA, all from the TAVI planning CT. *D–F* depict the corresponding images of the coronaries by invasive CA. The LAD was significantly stenosed. The patient underwent elective PCI of the LAD prior TAVI. CT, computed tomography; LAD, left anterior descending artery; LCX, circumflex artery; RCA, right coronary artery; TAVI, transcatheter aortic valve implantation.

At present, CA is the standard method for pre-TAVI CAD assessment.^17^ CA is generally safe; however, as with any invasive procedure, it can be associated with cardiac and non-cardiac complications that can range from minor issues to life-threatening events. Very recent data indicate the potential use of CTA in TAVI candidates (*Central Illustration*).^18,19^ A CT scan, including CTA, is an integral part of the general pre-TAVI workup, providing information about the anatomy of the aortic root and the aorta, the extent of vascular and valvular calcification, and the feasibility of vascular access.^20^ These CTA scans can also be used to evaluate the presence of significant CAD (*Figure 2*). However, the assessment of CAD on CTA has limitations in patients with severe AS as they often present with extensive coronary vessel calcification and heart rhythm disturbances and may not tolerate medications used to facilitate artefact-free CTA, such as nitroglycerin and beta-blockers (*Figure 3*). In addition, severe AS affects haemodynamics, potentially leading to suboptimal contrast timing during CT angiography. This can result in non-uniform enhancement of the coronary arteries, making it difficult to assess stenoses accurately. Severe AS can also cause beam hardening artefacts due to dense calcifications, further complicating image interpretation. However, technology is constantly improving, and such artefacts are rare.

Despite these limitations, several studies and meta-analyses have shown that CTA has an acceptable diagnostic accuracy for identifying significant CAD in TAVI candidates.^18,19,21^ These studies revealed an overall sensitivity of 90–97%, specificity of 65–92%, positive predictive value of 71–74%, and negative predictive value of 94–97%.^18,19,21^

CT even offers the use of fractional flow reserve (CT-FFR), which could provide both anatomical and functional assessments of coronary arteries. However, data regarding the use of this technology in AS patients are currently very scarce.^22^

Taken together, the use of CTA for the assessment of CAD in TAVI candidates would reduce risks associated with invasive CA such as vascular complications and contrast-induced nephropathy.^18,19^ It would furthermore shorten the time spent in hospital and potentially the waiting time from initial referral to TAVI.^8^ Since TAVI is expanding rapidly while resources remain limited, the utility of CTA to assess CAD during the pre-TAVI workup will certainly become increasingly relevant in the future.

## Revascularization of stable CAD at the time of TAVI

Apart from the TAVI setting, a proven prognostic benefit of coronary revascularization in stable CAD is limited to a few scenarios. The 2019 ESC guidelines on coronary revascularization indicated to revascularize stable CAD with significant left main or proximal left coronary artery stenosis (proximal segments as defined by the SYNTAX Score II), impaired left ventricular function, a large area of ischemia, or in the presence of a single remaining patent coronary artery for prognostic implications (Class IA). Conversely, in the more recent 2021 AHA guidelines,^23^ the prognostic indications for revascularization in stable CAD were downgraded and included only selected scenarios, such as left main disease.

So far, few studies have addressed the question of whether routine coronary revascularization at the time of TAVI is genuinely beneficial. Two randomized studies are currently available in that respect, the ACTIVATION trial (ISRCTN75836930)^2^ and the very recently published NOTION-3 study.^3^ The ACTIVATION, published in 2021, enrolled 235 patients who were randomly assigned to PCI of significant coronary stenoses before TAVI or to medical treatment of CAD. After 1 year, rates of death and rehospitalization were similar in both groups. However, PCI resulted in a significantly higher rate of bleeding complications. Although highly innovative and timely, the ACTIVATION was limited by several important factors: enrolment was long (2012–17), the average patient age was 84 years, bare metal stents were still in use, and anticoagulation of TAVI patients was more aggressive (dual antiplatelet inhibition generally recommended). Hence, the results of ACTIVATION are not fully transferable to today’s TAVI populations, particularly those with an intermediate- or low-risk profile. The just recently published NOTION-3 study^3^ enrolled 455 TAVI candidates (70% male, mean age 82 years) with at least one significant coronary artery stenosis (defined as FFR of 0.80 or less or a visual diameter stenosis of at least 90%) between 2017 and 2022. Patients were randomized to either undergo revascularization of all PCI-eligible lesions plus TAVI or TAVI alone and medical treatment of CAD. Patients with left main stem stenosis were excluded. Planned PCI after TAVI was not allowed in patients assigned to conservative treatment and the median SYNTAX score was only 9. After 2 years of follow-up (interquartile range, 1–4), the primary outcome, which was a composite of death from any cause, myocardial infarction, or urgent revascularization, occurred 10% more frequently in the conservative group (hazard ratio 0.71; 95% confidence interval 0.51–0.99; *P* = 0.04). This result was largely driven by more urgent revascularizations in the conservative group while no significant difference with respect to cardiovascular or overall mortality was documented. Bleeding events were more common in the PCI group. The trial’s results suggest that PCI for lesions with an FFR of ≤0.80 or a visual stenosis diameter of ≥90% should be considered for intervention in TAVI patients. However, the knowledge of untreated coronary lesions may have triggered unplanned revascularizations in the conservative treatment group, which is currently discussed as an important bias.^3^

Apart from ACTIVATION and NOTION-3,^2,3^ only retrospective data on this topic are available. Witberg *et al*.^4^ examined the association between CAD severity according to the SYNTAX score and all-cause mortality in 1270 consecutive TAVI patients. CAD was not significantly associated with mortality 2 years after TAVI. However, severe CAD and incomplete revascularization (rSS > 8) were associated with an almost two-fold risk of death, regardless of comorbidities. The authors concluded that a more complete revascularization of TAVI patients might attenuate the association between severe CAD and mortality. However, the study has important limitations: it was retrospective in design, represents a historical cohort (TAVI between 2009 and 2015), and was uncertain whether all patients with rSS > 8 could have been sufficiently revascularized. In addition, the authors did not provide information on the types of stents used or on follow-up medication. In another retrospective study, Li *et al*. demonstrated that rSS ≥ 10 was associated with increased rates of acute myocardial infarction and revascularization after TAVI. Nevertheless, overall mortality remained unaffected.^5^ The non-randomized retrospective REVASC-TAVI registry compared complete myocardial revascularization in TAVI patients with significant stable CAD with incomplete revascularization and showed no event-free survival benefit at 2 years.^24^ Even among predefined subgroups, which included young patients (<75 years), symptomatic patients, and patients with stenoses in proximal coronary segments or with left ventricular systolic dysfunction, the outcome remained unchanged. The optimal timing of revascularization in TAVI recipients has recently been addressed in a retrospective multicentre registry.^17^ The authors found that PCI after TAVI was associated with a better 2-year clinical outcome compared with other timing strategies (PCI before or concomitant with TAVI). The study groups had similar complexity and extent of CAD. The highest rates of acute kidney injury and in-hospital mortality were observed in the group with concomitant PCI and TAVI, and the lowest risk of bleeding was described in the group with PCI before TAVI. This registry is interesting and hypothesis generating but strongly limited by its retrospective and nonrandomized design.^1^

In several contemporary trials, only 5–13% of patients underwent PCI within 90 days of TAVI.^8,25^ The question of whether pre-emptive PCI of stable CAD in TAVI patients is beneficial at all is currently addressed by several randomized controlled studies. These include PRO-TAVI (NCT05078619)^26^ and COMPLETE TAVR (NCT04634240)^27^ (*Table 1*). All three trials randomize TAVI patients with significant CAD into complete revascularization vs. medical treatment of CAD. Completion is estimated between 2026 and 2028.

**Table 1:** Published and ongoing studies on CAD evaluation and management in TAVI patients

Study	Current status	Study design	Sample size (*n*)	Intervention	Primary endpoint
**Completed study**					
ACTIVATION^2^	Published 2021	Prospective randomized	310	PCI prior TAVI vs. medical therapy	Composite all-cause death and rehospitalization at 1 year
NOTION-3^3^	Published 2024	Prospective randomized open label	455	FFR-guided complete revascularization with PCI vs. medical therapy	Composite all-cause death, myocardial infarction, urgent revascularization at 2 years
**Ongoing studies**					
PRO-TAVI (NCT05078619)^26^	Recruiting	Prospective randomized open label	466	PCI prior TAVI vs. medical therapy	Composite all-cause death, myocardial infarction, stroke, major bleeding at 1 year
COMPLETE TAVR (NCT04634240)^27^	Recruiting	Prospective randomized open label	4000	Staged PCI (any time from 1 to 45 days post successful transfemoral TAVI) of all suitable coronary artery lesions ≥ 70% on visual assessment in vessels of >2.5 mm diameter vs. medical therapy.	Composite cardiovascular death, myocardial infarction, ischemia-driven revascularization, hospitalization for unstable angina, heart failure at a median follow-up time of 3.5 years
TAVI-PCI (NCT04310046)^28^	Recruiting	Prospective randomized open label	986	PCI 1–45 days before TAVI vs. PCI 1–45 days post-TAVI	Composite all-cause death, myocardial infarction, revascularization, rehospitalization, major bleeding at 1 year
FAITAVI (NCT03360591)^29^	Recruiting	Prospective randomized open label	320	PCI of lesions with FFR ≤ 0.80 vs. PCI of all lesions > 50% by visual estimation	Composite all-cause death, myocardial infarction, stroke, major bleeding, target vessel revascularization at 1 year

FFR, fractional flow reserve; PCI, percutaneous coronary intervention; TAVI, transcatheter aortic valve implantation.

Whether physiology-guided revascularization is beneficial in TAVI recipients is currently under investigation. One small nonrandomized retrospective study using FFR showed better MACCE-free survival at 24 months among those who underwent FFR-guided PCI compared with angiography-guided revascularization.^30^ Of note, FFR guiding yielded a significant reduction of the vessels requiring PCI. Following these preliminary data, FAITAVI^29^ and TAVI-PCI^28^ have been rolled out to focus on the optimized mode and extent of PCI in TAVI patients. TAVI-PCI randomizes patients to pressure-wire-guided complete revascularization within 40 days before vs. after TAVI,^28^ while FAITAVI compares visually with physiologically guided revascularization.^29^

Very limited data addressing the accuracy and safety of FFR, instantaneous wave-free ratio (iFR), and resting full-cycle ratio in TAVI candidates are currently available. Several limitations of such measurements have to be acknowledged. AS-associated left ventricular hypertrophy may cause changes in coronary flow physiology. Furthermore, AS may act like a tandem lesion, impacting both FFR and iFR measurements.^31^ In addition, the effect of intracoronary/intravenous adenosine may be blunted in AS patients, leading to underestimation of coronary stenosis severity with FFR.^32^ Studies that compared FFR measurements before and after TAVI found conflicting results. While some investigations showed a reduction in FFR after TAVI,^33,34^ an immediate FFR increase^35^ and no significant overall change in FFR were also described.^36^ For iFR measurements, which do not require adenosine and are independent of systolic flow, data are also inconsistent. While several studies found no changes in iFR immediately before and after TAVI,^32,34^ significant differences leading to a change in the indication for intervention in more than 25% of patients were also reported.^37^ Artificial intelligence-based CT-FFR has been evaluated for its ability to assess the functional significance of coronary lesions in 116 TAVI patients and revealed a 70% false positive rate. Most of these false positive CT-FFR readings indicating haemodynamically significant CAD were found in distal coronary segments. The authors explained this high false positive rate by an imbalance between the epicardial arterial volume and myocardial mass due to left ventricular hypertrophy.^38^

## ACS after TAVI

There are two reasons for considering coronary revascularization prior to TAVI: (i) to ensure the safety of the TAVI procedure and (ii) to avoid the need for future coronary intervention.

Generally, the most important driver for coronary intervention is acute coronary syndrome (ACS). Several studies have focused on the incidence and impact of ACS after TAVI. *Table 2* provides a list of recent publications.^5,39–44^ The rate of ACS after TAVI was consistently reported as being very low, with rates of 0.1–1.3% ST-elevation myocardial infarction (STEMI) and 2.4–5% non-ST-elevation myocardial infarction (NSTEMI) per year.

**Table 2:** Frequency and outcome of ACS after TAVI

Author study title	Study design	Population	Type of ACS (*n*)	Conclusion
Faroux *et al*.,^39^ *Acute coronary syndrome following transcatheter aortic valve replacement*	Prospective multicentre	Out of 6011 TAVI pts 270 (4.5%) ACS F/U 12 months	STEMI 22 (8.1%) NSTEMI 171 (63%) Unstable angina 77 (28.5%)	ACS was rare (<5%). Most ACS consisted of NSTEMI
Mentias *et al*.,^40^ *Incidence and outcomes of acute coronary syndrome after transcatheter aortic valve replacement*	Retrospective multicentre (US users of Medicare nationwide)	Out of 142 845 TAVI pts 6741 (4.7%) ACS F/U 10 months	STEMI 586 (8.7%) NSTEMI 5947 (88.2%) Unstable Angina 208 (3.1%)	ACS was rare (<5%), mostly NSTEMI. STEMI after TAVR associated with high mortality
Faroux *et al*.,^41^ *Procedural characteristics and late outcomes of percutaneous coronary intervention in the workup pre-TAVR*	Prospective multicentre	Out of 1197 TAVI pts with CAD, 100 (10%) ACS F/U 24 months	STEMI: 13 (13%) NSTEMI 62 (62%) Unstable angina: 25 (25%)	10% coronary event rate at 2 years, 1% STEMI
Nazir *et al*.,^42^ *Incidence, outcomes, and predictors of in-hospital acute coronary syndrome following endovascular transcatheter aortic valve replacement in the United States*	Retrospective National Readmission Database (USA)	Out of 48 454 TAVI pts 1332 (2.75%) ACS F/U 1 month	STEMI 152 (11%) NSTEMI 1180 (89%)	ACS was rare (2.8%) and mostly NSTEMI (89%). ACS pts were frail at baseline with multiple comorbidities. ACS after TAVI is associated with worse clinical outcome
Faroux *et al*.,^43^ *ST-segment elevation myocardial infarction following transcatheter aortic valve replacement*	Retrospective National Readmission Database (USA)	Out of 42 252 TAVI pts 118 (0.3%) STEMI F/U 8 months	STEMI 118 (100%)	STEMI was rare (0.3%) and associated with high mortality. PCI failure rate 17% after TAVI vs. 4% without TAVI
Li et al., 2019^5^ Impact of residual coronary atherosclerosis on transfemoral transcatheter aortic valve replacement.	Retrospective single centre	323 TAVI patients with CAD F/U 12 months	Information not provided	Patients with rSS ≥ 10 had increased rates of revascularization. Increased risk of MACE with rSS > 22.5. Overall mortality remained unaffected by rSS.
Vilalta *et al*.,^44^ *Incidence, clinical characteristics, and impact of acute coronary syndrome following transcatheter aortic valve replacement*	Prospective single centre	Out of 779 TAVI pts 78 (10%) ACS F/U 25 months	STEMI 1 (1.3%) NSTEMI 50 (64.1%) Unstable angina 27 (34.6%)	Completeness of pre-procedural revascularization did not predict ACS rate. ACS after TAVR associated with poor prognosis (3.8% mortality)
Tarantini *et al*.,^45^ *Coronary access and percutaneous coronary intervention up to 3 years after transcatheter aortic valve implantation with a balloon-expandable valve*	retrospective, European registry	Out of 1936 TAVI pts 68 (3.5%) CA (coronary angiography) F/U 3 years	STEMI 8 (11.8%) NSTEMI 18 (26.5%) Stable Angina 25 (36.8%) Dyspnoea 2 (2.9%) Chest pain 2 (2.9%) Syncope 1 (1.5%)	Coronary access successful in 100% of patients. PCI successful in 97.9% of patients

ACS, acute coronary syndrome; F/U, follow-up; MACE, major adverse cardiovascular event; NSTEMI, non-ST elevation myocardial infarction; pts, patients; rSS, residual Syntax score; STEMI, ST elevation myocardial infarction; TAVI, transcatheter aortic valve implantation.

Faroux *et al*.^39^ reported the important finding that most coronary events were related to new coronary lesions (not present or not considered significant at the time of CA prior to TAVI). It was also consistently shown that ACS was associated with poor in-hospital and late outcomes.^39,40,42–44^ In a large database analysis, Nazir *et al*.^42^ reported that TAVI patients who developed ACS were more frail at baseline with a higher comorbidity burden. Additionally, a history of PCI was identified as an independent predictor of ACS.

## Feasibility of PCI after TAVI

ACS requires urgent or emergent catheter-based coronary assessment and/or treatment. However, a TAVI prosthesis may significantly hinder percutaneous coronary access. The type of prosthesis and its geometry significantly impact coronary accessibility.^46^  *Table 3* lists the available literature regarding coronary access after TAVI, particularly regarding the impact of prosthesis type. Coronary access was excellent with the balloon-expandable Sapien valve, the Jena, and the Lotus Valve, while coronary access rates between 43% and 90% were reported for self-expanding platforms.^11,14^

**Table 3:** Feasibility of CA and PCI after TAVI

Study title	Study design	Population	Devices and results	ACS (*n*)
Gonçalves *et al*.,^47^ *Low rate of invasive coronary angiography following transcatheter aortic valve implantation: real-world prospective cohort findings*	Prospective single centre observational	Out of 563 TAVI pts 13 (2.3%) CA ± PCI F/U 24 months	9 pts, 11 PCIs PCI success: Balloon expandable 4/4 (100%) successful Self-expandable 3/7 (42.8%) successful, 2/7 (28.6%) partially successful, 1/7 (14%) unsuccessful	9
Nai Fovino *et al*.,^11^ *Incidence and feasibility of coronary access after transcatheter aortic valve replacement*	Prospective single centre observational	Out of 912 TAVI pts 48 (5.3%) CA ± PCI F/U 769 days	Coronary cannulation success: Balloon expandable 36/36 (100%) successful CoreValve (*n* = 6), Evolut Pro (*n* = 2): 6/8 (25%) RCA successful Jena Valve (*n* = 2): 2/2 (100%) successful Lotus Valve (*n* = 2) 2/2 (100%) successful	17
Tanaka *et al*.,^12^ *Incidence, technical safety and feasibility of coronary angiography and intervention following self-expanding transcatheter aortic valve replacement*	Prospective multicentre	Out of 2170 TAVI pts 41 (1.9%) CA ± PCI F/U 379 days	CoreValve. Evolut Pro CA: LCA: 28/32 (88%) successful, RCA: 16/32 (50%) successful PCI: 28/30 (93.3%) successful	29
Htun *et al*.,^13^ *Feasibility of coronary angiography and percutaneous coronary intervention after transcatheter aortic valve replacement using a Medtronic™ self-expandable bioprosthetic valve*	Retrospective single centre	Out of 403 TAVI pts 43 (11%) CA ± PCI F/U 15 months	CoreValve, Evolut R CA: LCA: 43/44 (97%) successful, RCA: 29/32 (91%) successful PCI: 29/29 successful (100%)	25
Zivelonghi *et al*.,^14^ *Coronary catheterization and percutaneous interventions after transcatheter aortic valve implantation*	Prospective single centre	66 CA of TAVI pts	Core Valve, Evolut R CA: 24/25 (96%) successful Edwards S3 41/41 (100%) successful	N/A

CA, coronary angiography; LCA, left coronary artery; PCI, percutaneous coronary intervention; RCA, right coronary artery; TAVI, transcatheter aortic valve implantation.

Good coronary access remains of paramount importance in young TAVI patients (<75 years), as future development of relevant CAD might be of prognostic significance. In patients in their eighties and nineties, the treatment of high-grade AS most likely represents the primary goal, also in the presence of CAD.

## Future directions

Taken together, the volume of TAVI procedures will certainly increase as population age. At the same time, available resources (doctors, nurses, catheter laboratories, and hospital beds) continue to be restricted.

It is, therefore, imperative that we sharpen our understanding of the role of routine CA and pre-emptive PCI of stable CAD in TAVI recipients.

Stable CAD remains one of the most frequent comorbidities among TAVI candidates. Although TAVI may be the preferred treatment for almost every AS patient in the future, the number of TAVI recipients aged 80 and above will remain high. In such elderly and often frail patients, CA carries significant risks while the benefits of CA are not well defined. The risk for ACS after TAVI is low (NSTEMI < 5% and STEMI < 1.3% in 2 years)^39–44^ and was shown to be not significantly influenced by pre-emptive revascularization.^48^ The NOTION-3 trial^3^ was the only study so far that demonstrated a benefit of revascularization of significant coronary stenoses in TAVI candidates. However, this result was driven by a reduction of revascularization during follow-up while mortality remained unchanged, in line with numerous studies demonstrating no prognostic impact of stable CAD that does not include the left main stem.^23^ At present, pre-emptive revascularization of stable coronary lesions at the time of TAVI remains common practice but ongoing randomized trials challenge the efficacy of this strategy.^21,26,27,49,50^ Routine invasive CA may frequently be unnecessary, at least if significant stenoses of the proximal coronary segments can be ruled out on the TAVI-planning CTA.^8,18,19,21,47,51^ A multicentre, multinational randomized study addressing such an algorithm is currently ongoing^50^ and will hopefully aid in paving the way for a more streamlined treatment flow for future TAVI patients.

## Data Availability

No new data were generated or analysed in support of this research. As this is an overview of the state of the art, all data are already listed in this paper.

## Lead author biography

Katharina Mascherbauer, born and raised in Vienna, Austria, is a medical doctor currently engaged in her residency training at AKH Vienna since 2023 and pursuing a PhD at the Medical University of Vienna since 2021. She completed her Human Medicine studies at the Medical University of Innsbruck (2014–21) and gained international experience during her studies in Internal Medicine at the University Hospital Zurich (2019), the Université Paris Descartes (2018), and Fundación Funavi Medical Center in Quito (2016). In research, she has been working in cardiology at the Medical University of Vienna since 2020, with a research focus on TAVI procedures and cardiac imaging. Besides the scientific career, she always pursued her volleyball career, with a history of several Austrian youth championships as team captain and she still plays among the top 5 teams in the Austrian Pro-Beach Tour.
